# The PDAC Extracellular Matrix: A Review of the ECM Protein Composition, Tumor Cell Interaction, and Therapeutic Strategies

**DOI:** 10.3389/fonc.2021.751311

**Published:** 2021-10-06

**Authors:** Vincent M. Perez, Joseph F. Kearney, Jen Jen Yeh

**Affiliations:** ^1^ Lineberger Comprehensive Cancer Center, University of North Carolina at Chapel Hill, Chapel Hill, NC, United States; ^2^ Department of Surgery, University of North Carolina at Chapel Hill, Chapel Hill, NC, United States; ^3^ Department of Pharmacology, University of North Carolina at Chapel Hill, Chapel Hill, NC, United States

**Keywords:** pancreatic ductal adenocarcinoma, extracellular matrix, tumor microenvironment, proteome, cancer

## Abstract

Pancreatic ductal adenocarcinoma (PDAC) is notorious for a dense fibrotic stroma that is interlaced with a collagen-based extracellular matrix (ECM) that plays an important role in tumor biology. Traditionally thought to only provide a physical barrier from host responses and systemic chemotherapy, new studies have demonstrated that the ECM maintains biomechanical and biochemical properties of the tumor microenvironment (TME) and restrains tumor growth. Recent studies have shown that the ECM augments tumor stiffness, interstitial fluid pressure, cell-to-cell junctions, and microvascularity using a mix of biomechanical and biochemical signals to influence tumor fate for better or worse. In addition, PDAC tumors have been shown to use ECM-derived peptide fragments as a nutrient source in nutrient-poor conditions. While collagens are the most abundant proteins found in the ECM, several studies have identified growth factors, integrins, glycoproteins, and proteoglycans in the ECM. This review focuses on the dichotomous nature of the PDAC ECM, the types of collagens and other proteins found in the ECM, and therapeutic strategies targeting the PDAC ECM.

## Introduction

With a 5-year survival of 10%, pancreatic ductal adenocarcinoma (PDAC) is the 3rd leading cause of cancer deaths in the United States and is projected to become the second leading cause of cancer deaths in the United States by 2030 (1, 2). Most patients have regional or distant spread at the time of diagnosis and are not eligible for a potentially curative operation (3). While advances have been made in the treatment of metastatic PDAC (4–6), most patients remain refractory to current regimens. Metastatic PDAC exhibits resistance to therapies like cytotoxic, radiation, and molecularly targeted therapies. A major factor thought to contribute to the treatment resistance of PDAC is its dense fibrotic stroma intertwined with the extracellular matrix (ECM), which together, provide a physical barrier of protection to the tumor cells and may also restrain tumor growth.

Research has shown that the ECM plays several roles beyond acting as a barrier for tumor cells. The deposition of abundant ECM proteins is common among solid tumors like PDAC and is known as a desmoplastic reaction (7). This tumor microenvironment (TME) exerts mechanical and biochemical properties on tumor cells, modulates interstitial fluid pressure (8, 9), and reduces blood vessel density within tumors (8). Although evidence has shown that the ECM may be tumor promoting (10), recent research has demonstrated that the stroma and ECM may restrain tumor growth (11, 12). In addition, tumor metastases, the primary cause of patient mortality, have less stroma than primary tumors (13, 14). A major regulator of the ECM are cancer-associated fibroblasts (CAFs), which dominate the TME in abundance (15).

CAFs are thought to arise predominantly from pancreatic stellate cells (PSCs) and bone-marrow derived mesenchymal stem cells. Their formation has been shown to be secondary to tumor signals (16). Several attempts have been made to target CAFs and the stroma; however, they have not been successful despite promising preclinical studies (9, 17). For example, genetic depletion of alpha-smooth muscle actin (αSMA) myofibroblasts in the stroma resulted in undifferentiated and invasive tumors with diminished survival (18). In metastatic PDAC patients, treatment with a pegylated recombinant human hyaluronidase (PEGPH20) combined with modified fluorouracil, leucovorin, irinotecan, and oxaliplatin (mFOLFIRINOX) did not improve progression-free or overall survival (19). Trials using hedgehog (Hh) pathway inhibitors in combination with gemcitabine showed that the addition of Hh inhibitors was not superior to gemcitabine alone, and one study was prematurely terminated as patients treated with the Hh inhibitor did worse than the control arm (20–22). Despite these failures, targeting and modifying CAFs and the ECM continues to remain a topic of interest due to the substantial roles they play in tumor development, growth, and metastasis (23).

The cross-regulated and interacting networks of ECM proteins are fundamental to tumor homeostasis and tumorigenic activity like growth and metastasis (24–27) and must be further understood. This dynamic environment provides a reservoir for signaling molecules and prompts tumor cell activity *via* mechanical forces and biochemical signaling. This review covers the composition and general roles and regulation of the ECM in PDAC, key proteins in the ECM, and potential and ongoing targeting strategies.

## ECM Overview

The ECM is a non-cellular component present in all tissue and provides the essential physical scaffolding, as well as biochemical and biomechanical cues to the surrounding cellular components. Consisting of water, proteins, and polysaccharides, the ECM composition adjusts based on the needs of the surrounding microenvironment. Most tissue require an ECM as an interstitial matrix in which the ECM is a 3-dimensional lattice supporting the surrounding cells of the stroma or a basement membrane in which the ECM is a matrix between epithelial and stromal layers of cells. Change in ECM architecture is a result of malignant cell transformation in many cancers, thus demonstrating the influence of biomechanical cues from the ECM (28, 29).

Tumor stroma has often been portrayed as a contributor to the development of the disease and tumor progression. However, several recent preclinical and clinical studies have shown that stroma-depletion can lead to a more aggressive disease (11, 18, 20, 30, 31). In patients, we and others have found that increased stroma correlates with improved survival and that stroma content at solid organ metastases is decreased (13, 14). Reduction of ECM through lysyl oxidase (LOX) inhibition in mouse models led to accelerated tumor growth (13).

**Figure 1:**
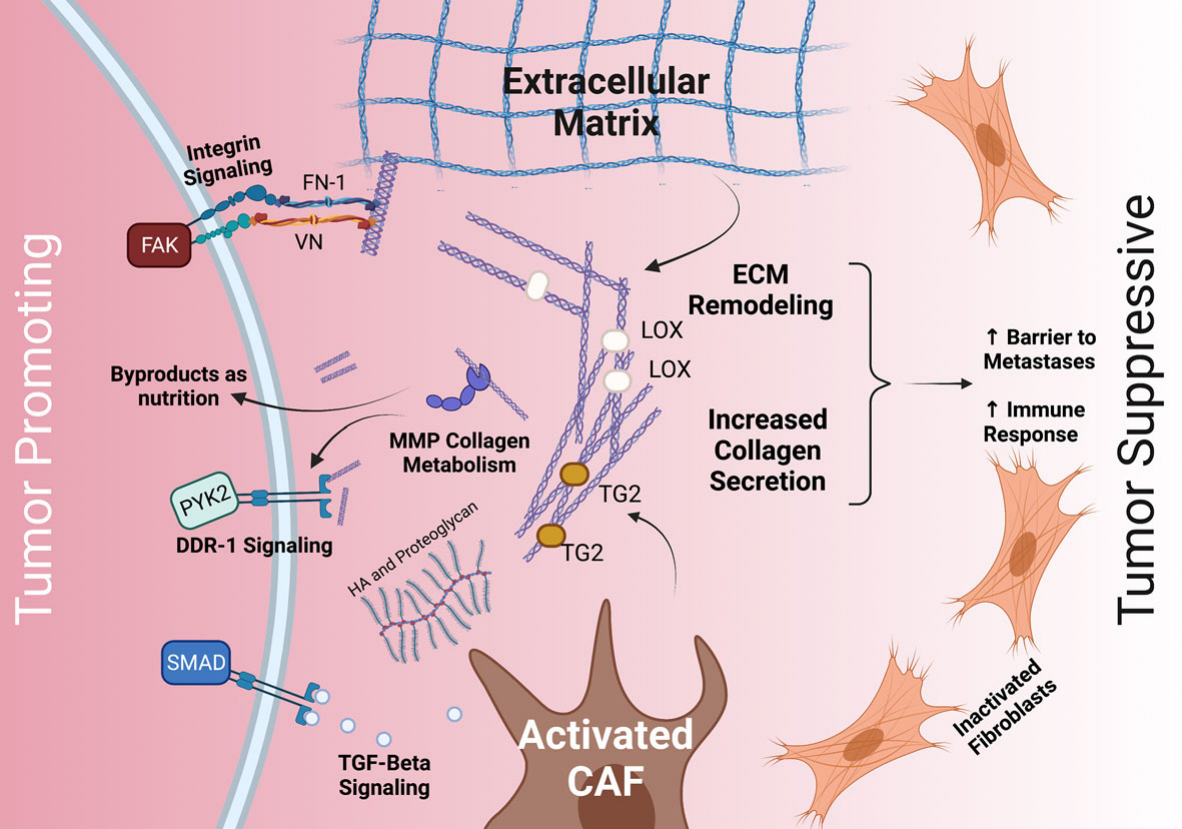
The dichotomous role of the stroma and common interactions between PDAC tumor cells, the ECM, and CAFs. The stroma’s ECM plays both a tumor promoting role (left) and a tumor suppressive role (right). Tumor promoting roles may include integrin, TGFβ/SMAD, and DDR-1 signaling, as well as providing nutrition *via* collagen fragments. Tumor suppressive roles may include providing a barrier to metastases and increasing immune response. The ECM (middle) is deposited by CAFs and modulated by MMP, LOX, TG2, HA, and others. CAF, cancer associated fibroblasts; TGF- beta, transforming growth factor beta; HA, hyaluronan; FN-1, fibronectin 1; VN, vitronectin; DDR-1, discoidin receptor 1; PYK2, FAK-related protein tyrosine kinase; FAK, focal adhesion kinase; TG2, tissue transglutimase; LOX, lysyl oxidase.

Thus, the stroma and ECM play a dichotomous role in the TME as a tumor-promoting and tumor-inhibiting component, and selectively targeting the stroma and ECM may be the key. However, a more complete understanding of the stroma and the TME is necessary for this to succeed. The PDAC ECM is primarily composed of collagens, integrins, proteoglycans, glycoproteins, and proteases which all interact with tumor cells through a variety of mechanisms (32, 33) (**Figure 1**). Among these, collagens are the most abundant component.

## Collagens

The PDAC ECM contains combinations of type I, III, IV, V, and XV collagens (32, 34, 35). The interaction between collagen and the basement membrane proteins within the ECM plays an important role during malignant transformation (36, 37). Increased desmoplasia is thought to contribute to disruption of the basement membrane leading to increased exposure of PDAC cells to interstitial collagens and reduction of basement membrane collagens (33). In PDAC, lower collagen I and IV is associated with undifferentiated PDAC tumors (38). Basement membrane proteins are suggested to be tumor-inhibiting by restricting the epithelial-to-mesenchymal transition (EMT), whereas interstitial collagens may promote tumor growth through other mechanisms. Preclinical studies have shown that collagen may contribute to therapy resistance by modulating signaling pathways (39–41). In patients, studies have found that imaging characteristics may be reflective of stroma content and patient outcome (14, 42).

### Collagens I, III, IV, and V

Collagen I is among the most abundant collagen in PDAC stroma and is generally suggested to be responsible for most of the desmoplastic reaction and has been associated with reduced survival in PDAC patients (43–45). However, when C-terminal prodomains of collagen I are cleaved by procollagen C-proteinase activity, collagen I may restrain tumor growth (46, 47). In addition, the presence of collagen I in the TME does not appear to inhibit T-cell infiltration (48), further suggesting the stroma has roles beyond a physical barrier. Collagen III is the second most abundant collagen present in the PDAC ECM (32). Like collagen I, collagen III may suppress tumor growth when cleaved (32). In addition, the ratio of collagen III to collagen III peptide fragments may be predictive of improved patient survival (49). Collagen IV, a collagen commonly found in the interstitial space, is overexpressed and deposited in the stroma by PDAC cells (50). *In vitro* assays using α1(IV)-siRNA demonstrated that collagen IV silencing reduces tumor cell proliferation and migration and increases apoptosis (51). Lastly, the fibrillar collagen, collagen V, is often identified as PSC-derived in PDAC and can be blocked by inhibiting β1-integrin signaling. Genetic knock-down of collagen V in orthotopic mouse models demonstrated reduced tumor metastasis and angiogenesis (52).

### Collagen Signaling

ECM collagens interact with signaling integrins expressed on the surface of PDAC cells. Collagens bind with the α- and β-subunits of integrins depending on the integrin specificity of the collagen subtype (53). Interstitial collagens I, IV, and V have a high affinity for integrin α2β1, and the interaction between collagen I or V with α2β1 promotes proliferation and migration of PDAC cells (51, 52, 54–56). Like integrin-mediated signaling, collagens also interact with the cell surface receptor discoidin receptor 1 (DDR-1), a tyrosine kinase overexpressed by PDAC cells. Active DDR-1 activates FAK-related protein tyrosine kinase (PYK2) (57).

### Collagen as Nutrients

Proliferating tumor cells require a constant supply of nutrients which are scarcely present in the dense desmoplastic tissue. Furthermore, the TME is generally hypoxic due to increased intra-tumoral oncotic pressure that decreases tissue perfusion. To adapt, PDAC cells may use large macropinosomes to non-specifically take up nutrients from the surrounding TME in bulk (58–60). In this way, PDAC cells are thought to actively scavenge the TME’s breakdown products and use collagen-derived proline as a primary source of energy in the absence of glucose (61, 62) (**Figure 1**). Likewise, proline oxidase (PRODH1) is overexpressed in proline catabolizing PDAC cells (62).

## Integrins, Proteoglycans, and Glycoproteins

In addition to collagen, the TME also contains integrins, proteoglycans, and glycoproteins. Integrins mediate the interactions between TME cell-types and ECM proteins like proteoglycans and glycoproteins. Both proteoglycans and glycoproteins undergo post-translational glycosylation which modulates their functions and conformation. Aberrant glycosylation, a hallmark of several cancers, modifies the behavior of proteoglycans and glycoproteins in the TME; therefore, it is expected that the two classes of proteins play several modified roles in the TME (63).

### Integrins

Integrins are transmembrane cell-surface receptors that heterodimerize upon binding ECM glycoproteins like fibronectin (FN1) and vitronectin (VN), collagens, and laminin (64). These cell-surface receptors are the major cell-adhesion receptors that interact with the ECM. In addition to directly contacting the ECM, activated integrins recruit the adhesome to the cytosolic tail of the integrin, a complex assortment of signaling, scaffolding, and cytoskeletal proteins (65–67). Differentially expressed in several solid tumors including PDAC, integrin roles are multi-faceted and participate in the metastatic cascade (68–70). Integrin-to-ECM contact points serve as molecular clutches to propel tumor cells forward by converting actin-polymerization into traction force (71, 72). In addition, by promoting the expression of matrix metalloproteinases (MMPs), integrins increase the proteolytic activity within the ECM and may facilitate the release of individual cancer cells or cell clusters from the TME (73). Integrin expression on cancer cells and endothelial cells has also been implicated in extravasation (74). In particular, PDAC zebrafish and mouse models demonstrated that the interaction between α5 integrin and neuropilin-2 facilitates the binding of cancer cells to the endothelium (75). Knockdown using lentiviral RNA-interference reduced cell adhesion, migration, and proliferation *in vitro* and *vivo* (76). In PDAC mouse models, the galectin-3 (GAL3) interaction with αvβ3 integrin may serve as an underlying mechanism of KRAS addiction and increase nutrient uptake *via* micropinocytosis (77). Altogether, PDAC has demonstrated overexpression of members of the integrin family like β1, β3, and β6 compared to normal pancreas (64, 69).

### Glycoproteins and Proteoglycans

The glycoprotein galectin-1 (GAL1) has been found to be upregulated in the PDAC TME, alongside other glycoproteins like periostin and fibulin and is lowly expressed in long-term (≥10 years) survivors of PDAC (78–80). Loss of GAL1 in murine models leads to reduced stromal activation and increased cytotoxic T-cell infiltration (81). FN1 and VN are thought to serve as scaffolding proteins in the PDAC TME and regulate cellular processes (82, 83). FN1 enables tumor cells to infiltrate the basement membrane, stimulates their proliferation, and bridges the interactions between ECM collagens and integrins (84–86) (**Figure 1**). FN1 is also known to stimulate transforming growth factor-β (TGFβ) secretion, in turn promoting the activation of the stroma (87). Secreted protein acidic and rich in cysteine (SPARC) is another major glycoprotein in the PDAC stroma that modulates ECM organization by directly binding to collagens I, III, IV, and V (88, 89). In addition, SPARC, expressed in the stroma from fibroblasts, regulates the interactions between integrins and ECM proteins by binding α5β1 focal adhesion sites (90). SPARC deficient mice demonstrated reduced collagen fibrillogenesis, and elevated metastasis (88).

The glycoprotein subclass, proteoglycans, often modulate the hydration level of the interstitial fluid, and in turn interstitial pressure, through its interactions with hyaluronic acid (HA) (91). However, HA modulation of interstitial pressure is suggested to be dependent on the collagen-richness of the TME and may not occur in collagen-replete TMEs (92). The TME of PDAC overproduces an abundance of HA beginning at the pre-malignant intraepithelial neoplasia (PanIN) stage (17). When HA is enzymatically depleted in mouse models, the delivery of cytotoxic therapies is enhanced, likely due to a compromised ECM lacking HA (17, 93).

### Secretome Studies

PDAC secretome analyses aim to examine the surrounding PDAC environment to identify aberrant proteins or molecules that may be influence tumor progression. A meta-analysis of 20 PDAC secretome studies from cell lines and patient tissues found that proteins associated with extracellular exosomes, blood microparticles, membrane-bound, secretory lumen and cytoplasmic membrane-bound vesicles, were most commonly found (94). Notable proteins found among the secretome and proteome across 20 secretome studies and 35 proteome studies include TGFβ induced (TGFBI), vimentin, and fibronectin (94). Using 3D organotypic cell cultures, Biondani et al. demonstrated that incubating cells with increasing type I collagen promoted the secretion of pro-angiogenic and growth factors like epidermal growth factor (EGF), matrix metalloproteinase 9 (MMP9), and vascular endothelial growth factor (VEGF) (95).

## Regulation of the ECM

PSCs are thought to maintain the balance between ECM synthesis and degradation and serve as a cellular reservoir for vitamin A and lipids (96). In the presence of tumor cells, PSCs assume an “activated” state in which they excessively deposit ECM proteins; become elongated in shape; and demonstrate increased expression of αSMA, collagens, immune-modulating, and other tumor-promoting genes (96). The formation of CAFs from PSCs is thought to be mediated by the PDAC secretome, which is abundant in fibroblast growth factor 2 (FGF2), TGFβ, and the paracrine and autocrine signaling regulator, sonic hedgehog (Shh) (97, 98). Shh functions to attract and activate PSCs, as PSCs demonstrate enhanced migration towards Shh overexpressing PDAC cells (98, 99). PSC activation through paracrine signaling has been shown to be inhibited with metformin, an activator of AMP-activated protein kinase (AMPK) (100).

TGFβ may suppress tumor growth in the early stages of carcinogenesis by inhibiting the proliferation of epithelial cells (101). However, in late stages of carcinogenesis, TGFβ promotes ECM deposition and tumor progression (101). A potent activator of PSCs, TGFβ mediates the interaction between the TME and tumor cells by binding TGFβ cell surface receptors. In PDAC, TGFβ promotes the deposition of several ECM proteins including fibronectin and collagens (102). On the surface of tumor cells, TGFβ receptor bind TGFβ to regulate downstream Smad-mediated gene transcription (103). PDAC-secreted TGFβ1 and FGF2 then promote ECM deposition by PSCs, as well as a positive feedback loop whereby PSCs secrete additional TGFβ1 that bind to TGFβ1-receptors found on the surface of PSCs. Once activated, PSCs, also known as CAFs, further modulate the ECM through a variety of mechanisms.

Tissue stiffness has been shown to enhance tumor cell proliferation in models of breast cancer (104). Stiff ECM matrices modulate vimentin, E-cadherin (CDH1), and ECM MMP activity (105, 106). Stiffening of the stroma is enhanced by collagen cross-linking carried out by LOX and tissue transglutaminase 2 (TG2) (107, 108) (**Figure 1**). LOX expression is increased under hypoxic conditions and systematic neutralization of LOX results in reduced proliferation and improved survival in mouse models (13, 107). On the other hand, TG2 secreted into the ECM promotes collagen I cross-linking and stimulates CAFs to produce additional collagen I (108, 109). In pancreatic tissue, TG2 is mildly expressed unless malignancy is present (108). Similar to stiffness, increased collagen fiber thickness is associated with poor patient outcome (110). The additional cross-linked collagen and stiff ECM, prompted by TG2 and/or LOX, activates yes-associated protein (YAP) and the transcriptional coactivator with a PDZ-binding motif (TAZ) that enhances cell proliferation (105). It is suggested that the YAP/TAZ signaling is the central hub for the cellular response to external mechanical cues, highlighted by increased nuclear localization of YAP/TAZ in response to mechanical stiffness (111–113).

## Targeting the ECM

### Hyaluronic Acid

The rationale for targeting the ECM as an adjunct to chemotherapy in PDAC relies on our understanding of the roles that ECM plays in promoting tumorigenesis. Early studies in mouse models showed that tumors with a high HA content had increased interstitial pressure that resulted in vascular collapse and diminished tumor perfusion. Treating these mice with a hyaluronidase inhibitor reversed these effects, creating an amenable environment for intra-tumoral drug delivery (17, 114). Since HA contributed to intra-tumoral pressure which in-turn affected drug delivery, the hypothesis was that targeting HA would improve drug delivery and efficacy. In SWOG S1313 (NCT01959139), PEGPH20 was evaluated in combination with mFOLFIRINOX and in the HALO trial (NCT02715804), in combination with nab-paclitaxel plus gemcitabine (**Table 1**) (19, 115). Unfortunately, some patients experienced strong adverse reactions to PEGPH20 and those who tolerated it did not have an improvement in overall survival (115).

**Table 1:** Selected clinical trials evaluating stroma and ECM targeting.

NCT registry number	Agent	Targets	Study population	Phase	Recruitment status
**NCT01959139**	PEGPH20 + modified FOLFIRINOX	HA + chemotherapy	Metastatic PDAC	I/II	Active, not recruiting
**NCT02715804**	PEGPH20 + nab-Paclitaxel + Gemcitabine	HA + chemotherapy	Hyaluronan-high Stage IV untreated PDAC	III	Terminated (sponsor decision)
**NCT01195415**	Vismodegib + Gemcitabine	Shh pathway + chemotherapy	Advance PDAC	II	Completed
**NCT01088815**	Vismodegib + Gemcitabine + nab-Paclitaxel	Shh pathway + chemotherapy	Metastatic PDAC	II	Completed
**NCT01064622**	Vismodegib + Gemcitabine	Shh pathway + chemotherapy	Recurrent or metastatic PDAC	I/II	Completed
**NCT01383538**	IPI-926 + FOLFIRINOX	Shh pathway + chemotherapy	Advance PDAC	I	Completed
**NCT01585701**	AT13148	Muti-AGC kinases including ROCK-AKT	Advance solid tumors	I	Completed
**NCT03519308**	Paricalcitol + Nivolumab + nab-Paclitaxel + Gemcitabine	Vitamin D Receptor + PD-L1 + chemotherapy	Resectable PDAC	I	Recruiting
**NCT04617067**	Paricalcitol + Gemcitabine + nab-Paclitaxel	Vitamin D Receptor + chemotherapy	Advance PDAC	II	Recruiting
**NCT04524702**	Paricalcitol + Hydroxychloroquine + Gemcitabine + nab-Paclitaxel	Vitamin D Receptor + autophagy + chemotherapy	Advance or metastatic PDAC	II	Recruiting
**NCT03331562**	Paricalcitol + Pembrolizumab	Vitamin D Receptor + PD-1	PDAC (maintenance)	II	Completed
**NCT02930902**	Paricalcitol + Pembrolizumab + Gemcitabine + nab-Paclitaxel	Vitamin D Receptor + PD-1 + chemotherapy	Resectable PDAC	I	Active, not recruiting
**NCT02030860**	Paricalcitol + nab-Paclitaxel + Gemcitabine	Vitamin D Receptor + chemotherapy	Resectable PDAC	NA	Completed
**NCT03138720**	Paricalcitol + nab-Paclitaxel + Gemcitabine + Cisplatin	Vitamin D Receptor + chemotherapy	Advance PDAC	II	Active, not recruiting
**NCT03415854**	Paricalcitol + nab-Paclitaxel + Gemcitabine + Cisplatin	Vitamin D Receptor	Metastatic PDAC	II	Active, not recruiting
**NCT02754726**	Paricalcitol + Nivolumab + nab-Paclitaxel + Gemcitabine + Cisplatin	Vitamin D Receptor + PD-L1 + chemotherapy			Active, not recruiting
**NCT01646203**	LY3022859	Tβrii	Advance solid tumors	I	Completed
**NCT00557856**	PF-03446962	Tβri	Advance solid tumors	I	Completed
**NCT04296942**	Bintrafusp alfa	Tβrii + PD-L1	Advance solid tumors	I	Completed
**NCT01682187**	Galunisertib	Tβri	Advance solid tumors	I	Completed
**NCT01373164**	Galunisertib + Gemcitabine	Tβri + chemotherapy	Inoperable or metastatic PDAC	I/II	Completed
**NCT02154646**	Galunisertib + Gemcitabine	Tβri + chemotherapy	Inoperable or metastatic PDAC	I	Completed
**NCT02734166**	Galunisertib + Durvalumab	Tβri + PD-L1	Metastatic PDAC	I	Completed
**NCT03192345**	SAR438459 + Cemiplimab	Tgfβ1, tgfβ2, and tgfβ + PD-L1	Advance solid tumors	I	Active, recruiting

### Hedgehog Pathway

Treating mice with Shh inhibitors in addition to chemotherapy improved tumor microvascular density and survival (9). The addition of Shh inhibitors to either FOLFIRINOX or gemcitabine in clinical trials failed to improve overall survival and, in some cases, was associated with a worse outcome (**Table 1**) (20, 30). These studies were done prior to our understanding of CAF heterogeneity, and recent work has been done to further elucidate the underlying biology responsible for the discrepancy in clinical findings. Recent studies suggest that blocking the Shh signaling pathway may shift the stroma CAF populations from myofibroblast-CAFs (myCAFs) to inflammatory CAFs (iCAFs) and promote an immunosuppressive TME (116).

### Rho Kinase Inhibitors

Rho kinase (ROCK) inhibitors alter PDAC cell cytoskeletal contractility and CAF contractility and may enable more favorable drug delivery in PDAC as well as inhibit PDAC metastasis. ROCK inhibition resulted in a disorganized ECM that blunted PDAC migration and invasion *in vitro*. *In vivo* models including both subcutaneous and intrasplenic injection found that administering ROCK-inhibitors prior to chemotherapy resulted in increased primary tumor response to chemotherapy and helped prevent the growth and establishment of liver metastases (117–120). A phase 1 study using a kinase inhibitor, AT13148, with anti-ROCKI/II activity was carried out in patients with metastatic solid tumors, but did not proceed further due to toxicity (**Table 1**) (121).

### Vitamin D

Vitamin D halts the secretion of collagen in several cell types, possibly by disrupting TGF-β signaling (103, 122). The vitamin D receptor (VDR) has been shown to regulate transcription of PSCs and CAFs (123, 124). Treatment with VDR ligand, calcipotriol, reprograms the stroma, decreases inflammation and improves response to gemcitabine (123). Other studies have shown that while calcipotriol decreases CAF migration and inflammation, it also may negatively affect T cell effector functions (125, 126). Several studies of paricalcitol, a vitamin D analogue, in combination with chemotherapies and immunotherapies are ongoing (**Table 1**).

### TGFβ

Preclinical experimental data has suggested that blocking TGFβ-mediated signaling likely enhances antitumor effects in solid tumors. Further, PDAC is often characterized by high TGFβ expression levels (101). Several clinical trials have attempted to target TGFβ in a variety of cancers, but no TGFβ-targeting inhibitor has been approved for use (127). There is currently at least one ongoing clinical trial targeting TGFβ in PDAC (**Table 1**) that is investigating the pharmacokinetics, pharmacodynamics, and anti-tumor activity of SAR439459 alone and in combination with cemiplimab. SAR439459 is a pan-TGFβ neutralizing antibody that targets the three TGFβ isoforms (TGFβ1, 2 and 3) and their interaction with type II TGFβ cell surface receptors (128).

## Concluding Remarks

The ECM plays an important role in PDAC tumor growth, metastasis, and therapy resistance. Accumulating preclinical studies with patient-derived specimens indicate that targeting the dense desmoplastic ECM proteins of PDAC may provide promising clinically useful therapies. In clinical practice, we have yet to successfully target the ECM in a way that improves overall survival. The clinical shortcomings of once promising therapies are a humble reminder of the underlying complexity of tumor biology. Learning from these failures and developing a deeper understanding of the fundamental biology allows researchers to identify further vulnerabilities that should be considered when developing treatments. Tumor and CAF subtypes have been identified that were previously not known and will likely improve the outcome of ECM modulating studies if performed using reproducible subtyping methods and tailored targeting techniques (15, 16, 129–133). Recent advances foster hope that ECM-modulating therapies are likely to become a crucial part of the toolkit for oncologists in the next few decades.

## Author Contributions

All authors listed have made a substantial direct and intellectual contribution to the work and approved it for publication.

## Funding

This work was financially supported by the National Cancer Institute of the National Institutes of Health under the award numbers R01-CA199064 (JY) and T32-CA244125 (JK, VP).

## Conflict of Interest

The authors declare that the research was conducted in the absence of any commercial or financial relationships that could be construed as a potential conflict of interest.

## Publisher’s Note

All claims expressed in this article are solely those of the authors and do not necessarily represent those of their affiliated organizations, or those of the publisher, the editors and the reviewers. Any product that may be evaluated in this article, or claim that may be made by its manufacturer, is not guaranteed or endorsed by the publisher.
